# Evaluation of Mechanical Properties and Volatile Organic Extractable to Investigate LLDPE and LDPE Polymers on Final Packaging for Semisolid Formulation

**DOI:** 10.3390/pharmaceutics10030113

**Published:** 2018-08-02

**Authors:** Arianna Cecilia Cozzi, Benedetta Briasco, Enrico Salvarani, Barbara Mannucci, Filippo Fangarezzi, Paola Perugini

**Affiliations:** 1 Department of Drug Sciences, University of Pavia, Via Taramelli 12, 27100 Pavia, Italy; ariannacecilia.cozzi01@universitadipavia.it (A.C.C.); benedetta.briasco@gmail.com (B.B.); 2 Lameplast (Joint-Stock Company), Via Verga 1-27, Rovereto s/S, 41016 Novi di Modena, Italy; enrico.salvarani@lameplast.it (E.S.); filippo.fangarezzi@lameplast.it (F.F.); 3 Centro Grandi Strumenti (Center for Large Equipment), University of Pavia, Via Bassi 21, 27100 Pavia, Italy; barbara.mannucci@unipv.it

**Keywords:** packaging evaluation, mechanical properties, extractable testing, content-container interaction, LDPE, LLDPE

## Abstract

Plastic material is used for a wide variety of commercial packaging due to being inexpensive, lightweight, and due to its resistance. In pharmaceutics, container-content compatibility studies are required for product authorization. Many guidelines and publications are available; however, the information is often only related to the raw materials used to produce packaging. During the manufacturing process, substances can be added to improve the product characteristics and performance, resulting in a processed material that is considerably different from the unprocessed material. In this study, the mechanical properties of low-density polyethylene (LDPE) and linear low-density polyethylene (LLDPE) specimens fabricated according to standard ISO 527 and specimens fabricated with the same materials, but obtained from final packaging, were evaluated. Furthermore, we examined the interaction between a semisolid formulation and LLDPE and LDPE as a final packaging, by subjecting two samples to accelerated degradation testing. Then, mechanical properties and volatile organic extractable were evaluated. Simulated solar radiation did not induce changes in the packaging mechanical properties and no extracts were detectable. The thermal shock strongly influenced the mechanical behavior, and interactions between packaging contents were identified. The present work underlines the difference between analyzing the standard ISO specimens versus samples obtained from final packaging in order to evaluate the packaging under real use conditions. An evaluation on the final packaging, instead on standard specimens, can provide information about the plastic material after the manufacturing process and the interaction between packaging and content.

## 1. Introduction

Packaging plays a key role in preventing spoilage, extending shelf life, and facilitating storage and transport, but the packaging has to fulfil more than these primary containment, preservation, and protection functions. Packaging also influences the sale of products with a promotional function, delivering information about the convenience and outlining claims about the final products [1,2,3,4]. Even if the packaging should preserve and protect the content from external physical, chemical, and microbiological hazard in order to maintain the safety, quality, and effectiveness of the product, studies about the interactions between the packaging material and the product contained have highlighted possible migration of chemical substances across the packaging sourced from the contained or the sorption of product ingredients by the packaging. Moreover, the loss of volatile compounds or the diffusion of volatiles from the environment (O_2_, CO_2_) toward the contents could result in degradation (e.g., oxidation) or a microbiological contamination [5,6,7,8,9,10,11].

Pharmaceutics packaging involves two critical aspects: the product’s quality and the safety. Quality is evaluated using a stability study performed on the product; safety is partially evaluated with a stability study and toxicity testing. The toxicity is the key focus of the container-content compatibility study and for patient safety. For cosmetic products, Regulation 1223/2009 commits to reporting information about “impurities, traces, and information about the packaging material” [12]. However, no guidelines are currently available for testing cosmetic packaging. Testing is only performed on packaging materials used for food. The food sector is regulated by European Regulation No. 1935/2004 for materials and articles intended to contact food, and Commission Regulation (EU) No. 10/2011 is specific to food-contact plastic materials. This regulation establishes a list of compounds authorized for use in plastic formulation and migration tests performed on food simulants because the packaging and the contained product are not two separated entities, but they may interact, especially in presence of varying environmental conditions. Penetration of content components into the packaging or migration of packaging substances into the product could produce significant variation in the packaging properties or affect product safety and efficacy.

One of the main and often measured properties of plastic materials used in structure applications is the ability to resist breaking under tensile stress. Tensile testing provides information about yield strength, ultimate tensile strength, modulus of elasticity, and elongation. It is a reliable method used to obtain data about how different conditions (exposure to various temperature and humidity conditions, ultraviolet (UV) radiation, etc.) may affect the performance of the final product. Universal testers are available: International Organization for Standardization (ISO) system (ISO 527) and the ASTM (American Society for Testing and Materials) system (D638) [13,14]. Usually, this testing is performed on standardized specimens. During the manufacturing process, substances such as plasticizers, thermal stabilizers, lubricant, light stabilizers, and pigments are added to improve product characteristics and performance. These additives have been shown to alter the material characteristics [15,16,17]. As such, the unprocessed material could be considerably different from the final packaging, underling the importance of a valuation on the plastic material obtained from the final plastic packaging and not from the unprocessed material or a standardized specimen.

In this paper, low-density polyethylene (LDPE) and linear low-density polyethylene (LLDPE) were selected as plastic materials. They are common plastic materials used for flexible bags, battels, single-dose, caps, blister packs, etc. [18,19,20], widely applied for their suitable production characteristics.

## 2. Materials

Xanthan gum transparent 8 mesh (ACEF SpA, Fiorenzuola d’Arda, Italy), sodium bicarbonate, borax, 37% hydrochloric acid, sodium hydroxide pellet (CARLO ERBA reagents, Cornaredo, Italy). Dimethicone KF995 (Prodotti Gianni srl, Milan, Italy). Tegosoft diethylhexyl carbonate (DEC) and Abil CARE XL 80 (Bis-PEG/PPG-20/5 PEG/PPG-20/5 Dimethicone; Methoxy PEG/PPG-25/4 Dimethicone; Caprylic/Capric Triglyceride) (Evonik Industries, Essen, Germany). Type 2 purified water obtained from the Milli-Q^®^ purification system (Merck, KGaA, Darmstadt, Germany) was used.

### 2.1. Polymeric ISO Specimens

ISO (ISO 527-1:1996) specimens of low density polyethylene (LDPE-ISO) were provided by Lameplast S.p.A. (Rovereto s/S, Novi di Modena, Italy) from polymer pellets (Purell 1840) provided by Lyondell Basell (Lyondell Basell, Rotterdam, The Netherlands).

ISO (ISO 527-1:1996) specimens of linear low density polyethylene (LLDPE-ISO) were provided by Lameplast S.p.A. (Rovereto s/S, Novi di Modena, Italy) from polymer pellets (Stamylex 2258) provided by DEXplastomers (DEXplastomers, Borealis, The Netherlands).

### 2.2. Final Packaging

Single dose containers (5 mL) of low-density polyethylene (LDPE) were provided by Lameplast (Lameplast, Rovereto s/S, Novi di Modena, Italy), and 5 mL single dose containers of linear low-density polyethylene (LLDPE) were provided by Lameplast (Lameplast, Rovereto s/S, Novi di Modena, Italy).

## 3. Methods

### 3.1. Simulant Production and Characterization

In order to detect some criticities in semisolid formulation, such as high pH and silicone presence, an appropriate emulsion was prepared. Silicone chemistry plays a key role in personal care and cosmetic formulations due to a multifunctional set of properties [21,22]. Table 1 shows the qualitative and quantitative composition of the formulation intended to fill the final packaging and used as the simulant. Phases A and B were stirred separately. Phase B was added to phase A using a Silverson SL2T High Shear Laboratory Stirrer Mixer (Silverson Machines Ltd., Chesam, UK) for 10 min, 6700 rpm, and at 50 °C.

The formulation was stored overnight at 25 °C. Successively, pH organoleptic characteristics and rheological properties were evaluated. The pH measurement was performed using a pH meter model 3510 (Jenway, Staffordshire, UK), and viscosity properties were evaluated using a Kinexus Pro+ rheometer (Malvern, Worcestershire, UK), equipped with Peltier Plate Cartridge, with cone geometry CP40/4.

### 3.2. Accelerated Stability Testing

The final packaging, both empty and filled with simulant, were subjected to accelerated degradation testing in order to simulate the “in use” stress conditions that the final product could enco0unter during its life. Simulated solar irradiation and thermal shock cycles were used.

#### 3.2.1. Simulated Solar Irradiation

Simulated solar irradiation was performed using SUNTEST XLS + II (Atlas^®^, Chicago, IL, USA) according to standard European procedures [23] with the following parameters: irradiation control 300–800 nm, irradiation 750 W/m^2^, room temperature 35 °C, and black standard temperature (BST) 45 °C. The samples were irradiated for 48 h on each side of the final packaging, for a total of 96 h of irradiation.

#### 3.2.2. Thermal Shock Cycles

Thermal shock cycle testing was performed in the Clima Cell 111 MMM Medcenter Einrichtungen GmbH climatic room, Munchen, Germany. The samples were exposed to 4 °C for 7 days and then to 37 °C for other 7 days. This cycle was repeated twice.

### 3.3. Mechanical and Migration Tests

After accelerated stability testing, the final packaging filled with simulant was emptied and washed with 1% bicarbonate solution and rinsed with distilled water in order to eliminate the simulant excess. The same treatment was performed on the final empty packaging to maintain the same experimental conditions. Those samples were subjected to mechanical tests and evaluation of extractables and then compared with controls.

#### Mechanical Test

We investigated the mechanical properties using a tensile machine, AGS 500 ND (Shimadzu Corporation, Kyoto, Japan) equipped with a 500 Newton (N) load cell, with a crosshead stroke of 1100 mm, and tensile stroke of 740 mm. The test was performed using a strain rate of 10 mm/min. The test protocol for the mechanical measurements was reported in a previous work [24]. Estimating the following is possible: elastic portion by a linear regression procedure (Et); stress properties: yield stress (σy), tensile strength (σM), and tensile stress at break (σB); and tensile strain expressed as the increase in length: at yield (εy), at tensile strength (εM), and at break (εtB). From each set of results, we estimated the tendency of materials to oppose deformation, and evaluated the curve profile in elasticity regime, the elongation percentage in the elasticity regime, and the absolute elongation elasticity.

### 3.4. Tensile Test Specimens

A total of 25 dog bone shaped specimens, for each kind of container, were obtained horizontally from the central part of the 5 mL untreated and treated single dose containers. We made this choice because the small size of the containers did not allow us to obtain vertical specimens. An optimized dog bone shape was obtained by punchcutting as described in a previous work [24]. We created the specimens in accordance with European Standard EN ISO 527 [25].

The only standard reference found for tensile testing on plastic material was ISO 527. ISO 527 standard specimens are created by injecting the melted polymeric pellets in a standard mold. In the considered ISO, no information about the injection region was found. Consequentially, 10 specimens for each material (LDPE and LLDPE) were obtained by molding following the ISO 527 standard: 5 ISO specimens for each material were created with a polymeric injection from the side of the mold (H-ISO), and 5 ISO specimens for each material were created with a polymeric injection from the bottom of the mold (V-ISO). Using this method, horizontal and vertical polymeric chain alignments were considered in the production, and two types of specimens were obtained: horizontal (H-ISO) and vertical (V-ISO) specimens, respectively.

Each specimen was characterized in terms of thickness and width of this region using a digital microscope, model BW1008-500x (Farnell element14 Trade Counter, Leeds, UK). The section of each sample was calculated from thickness and width using a suitable software program (Micro-Measure version 1.2, Colorado State University, Fort Collins, CO, USA).

### 3.5. Statistical Analysis

The data obtained from the mechanical test on specimens were processed via the Mann-Whitney test and comparing specific tests for parametric and nonparametric data. We chose a confidence range of 95%, so the changes were considered statistically significant for *p* < 0.05.

### 3.6. Extractable Testing

In order to evaluate the extractable profiles, plastic materials before and after specific treatments were subjected to different extraction conditions and the resulting extractions were analytically characterized by gas chromatography-mass spectrometry (GC/MS) to establish the material profile of each extracted volatile organic compounds. An optimization of the controlled extraction method was studied. This test procedure could provide a good baseline to determine a method for controlled extraction studies specifically relevant for the plastic materials investigated.

### 3.7. Extraction Methods

Multiple extraction processes were evaluated to maximize the predominant extractable. The extraction process and extraction solvents were chosen in relation to the plastic materials investigated and according to the literature [26]. Table 2 shows the specific of extraction methods used: sonication, Sealed Vessel, Soxhlet, and Head Space Solid Phase microextraction (HS-SPME). Extraction solvents included: low pH water buffer solution pH = 2, high pH water buffer pH = 10, 1:1 isopropanol/water mixture, and hexane. All extraction processes were conducted in duplicate. Table 2 provides the extraction methods specifications.

### 3.8. Characterization by Gas Chromatography-Mass Spectrometry (GC/MS)

The extracts obtained from the extraction method were characterized by gas chromatography-mass spectrometry (GC/MS). Analyses were performed by using a Thermo Fisher Scientific, (Waltham, MA, USA) GC/MS system (TraceDSQII mass spectrometer, TraceGCUltra gas chromatograph, CTC Analytics COMBIPAL autosampler), and Xcalibur MS Software Version 2.2. Operating parameters are reported in Table 3. The mass spectra of the detected extractable compounds were compared with the GC/MS NIST Mass Spectral Library (NIST 08) databases and the 8th Edition Wiley Registry of Mass Spectral Data. Although databases were used, some classes of compounds, such as alkanes, yielded similar fingerprint patterns or fragments, and thus we were not always able to cleanly identify every peak (compound) detected.

## 4. Results

### 4.1. Tensile Test Specimens

Thickness of the LLDPE and LDPE specimens created according to ISO 527 specifications [14] and LLDPE and LDPE dog-bone shaped specimens obtained from the final single-dose packaging containers were calculated to determine if the specimens were uniform. Table 4 shows the thickness means obtained.

The tensile test was performed on the samples. Table 5 shows tensile strength (σM), tensile strain at yield (εy), angular coefficient linear portion, tensile stress at break (σB), and tensile strain at break (εtB) data obtained from the tensile test on the ISO and dog-bone shaped specimens expressed as a mean ± standard deviation (%).

**Figure 1:**
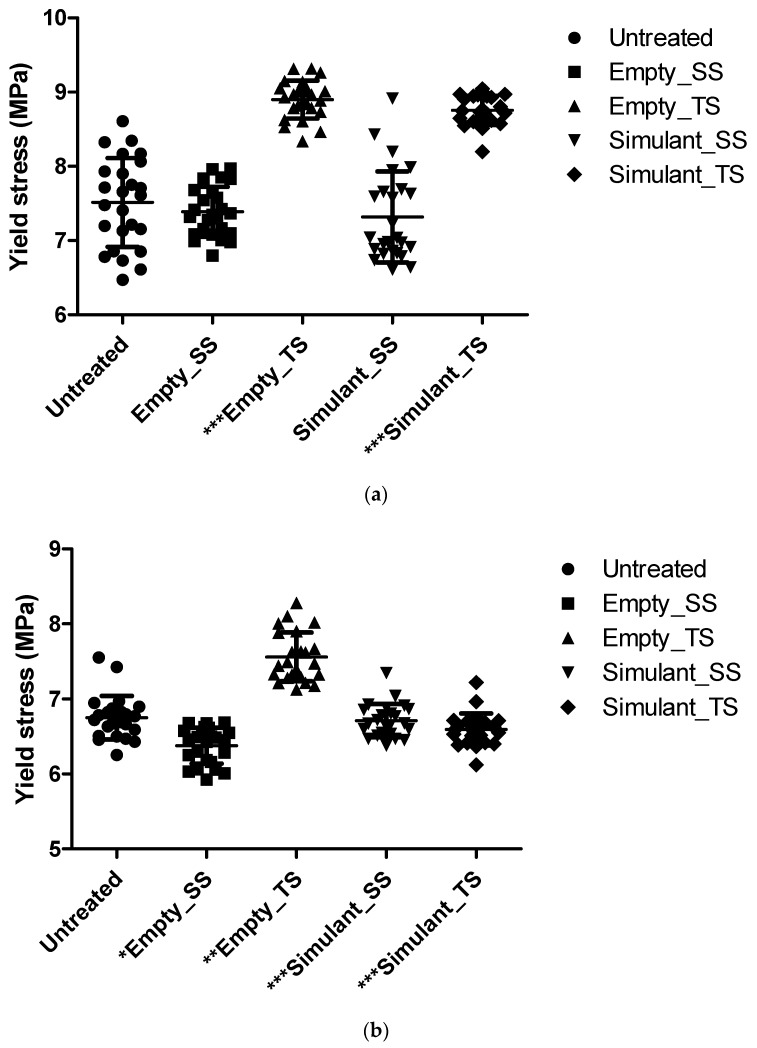
Trend of yield stress values for containers filled with simulant after simulated solar irradiation (SS) or thermal shock (TS): (**a**) Linear low-density polyethylene (LLDPE) packaging (statistical significance: *** *p* < 0.0001 compared to Untreated); (**b**) Low-density polyethylene (LDPE) packaging (statistical significance: * *p* = 0.2 compared to Untreated; ** *p* = 0.025 compared to Untreated; *** *p* < 0.0001 compared to Untreated).

Single-dose LLDPE and LDPE containers, either empty or filled with simulant, were subjected to accelerated stability testing. A total of 25 dog-bone shaped specimens of each sample were analyzed with the tensile test. A graph representing the yield point value trend for LLDPE and LDPE are reported in Figure 1.

### 4.2. Extract Characterization

The volatile organic extractable profile of the plastic materials obtained from untreated final packaging was investigated using multiple extraction processes. Extraction solvents were used in order to detect the technique able to identify the major class of components in the plastic material.

**Figure 2:**
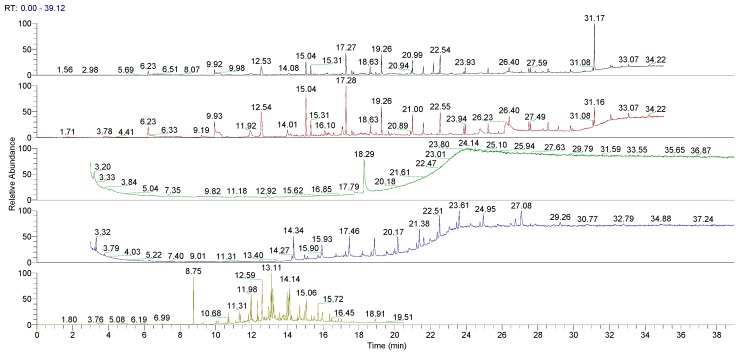
Chromatograms obtained by gas chromatography-mass spectrometry (GC/MS) of all the extraction methods on untreated linear low-density polyethylene (LLDPE) single dose containers. From the top: Sonication pH 2, sonication pH 10, Sealed Vessel, Soxhlet, and HS SPME (Head Space Solid Phase microextraction).

**Figure 3:**
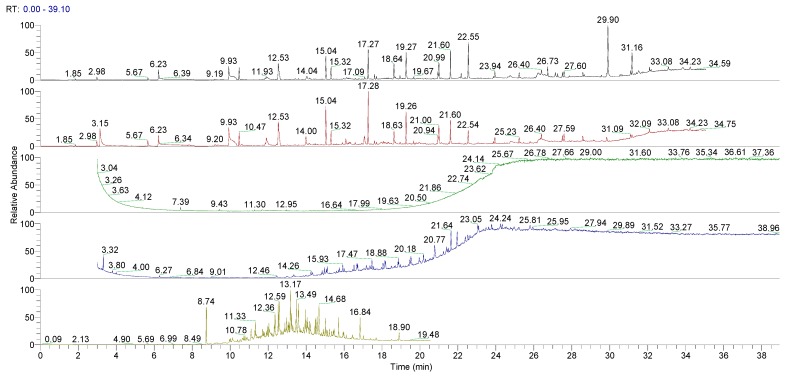
GC/MS chromatograms obtained of all the extraction methods for untreated low-density polyethylene (LDPE) single dose containers. From the top: Sonication pH 2, sonication pH 10, Sealed Vessel, Soxhlet, and HS SPME (Head Space Solid Phase microextraction).

Figure 2 and Figure 3 show the Total Ion Current (TIC) chromatograms related to the GC/MS analysis of the various extracts obtained from untreated LLDPE and LDPE single dose containers.

After subtraction of the extraction blanks from the samples and removal of the interfering peaks through bleeding the GC capillary column or SPME fiber coating, more than 100 substances were identified. For simplification, the HS-SPME extraction method associated with GC/MS analysis was chosen. The detected substances were divided into three different categories: (1) compounds associated with the initial ingredients (e.g., antioxidants, additives, and amides); (2) impurities related to processing (e.g., oligomers, residual solvents, esters, and siloxane); and (3) degradation products of polymers (e.g., fragments of saturated and unsaturated hydrocarbons, ketones, and acids), as previously reported [27]. Table 6 shows some substances detected from untreated LLDPE and LDPE single dose containers.

LLDPE and LDPE single dose containers, both empty and filled with simulant, were characterized after an accelerated stability test. Extractable profiles of the treated samples were created via HS-SPME extraction and the extracts were analyzed by GC/MS. Table 7 presents the percent area of each class of extracted substances.

**Figure 4:**
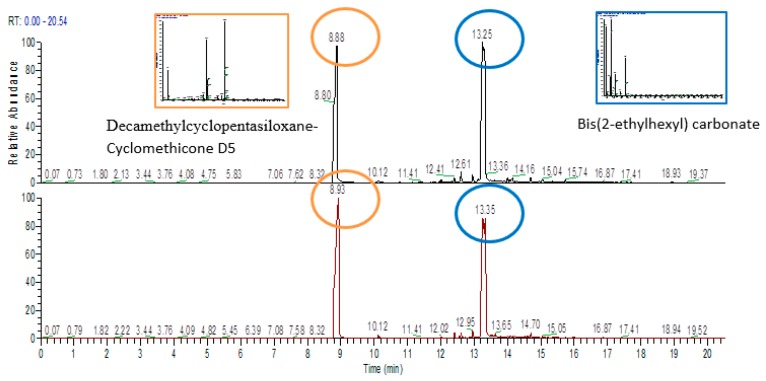
Chromatograms for (**top**) linear low-density polyethylene (LLDPE) and (**bottom**) low-density polyethylene (LDPE) single dose containers filled with simulant after simulated solar irradiation. (RT: retention time expressed in minutes).

After simulated solar irradiation or thermal shock of the filled samples, substances closely related to the simulant were detected in the extractable profile of the plastic material. These substances were identified as Decamethylcyclopentasiloxane-Cyclomethicone D5 and *Bis*(2-ethylhexyl) carbonate, and they represented nearly 95–96% of the total extracted compounds. Figure 4 shows chromatograms of the LLDPE and LDPE single dose containers filled with simulant after simulated solar radiation.

In order to exclude the possibility that the identified substances simply remained on the surface of the polymers in a non-efficient washing system, LLDPE and LDPE samples from the final packaging were placed in the simulant for 30 min. Then, they were cleaned with the washing method used for all the samples. An extractable profile, with the selected test technique (HS-SPME), was created. After GC/MS analysis, no traces of the substances associated with the simulant were found. Successively, a preliminary study was completed to evaluate the substances realized from the final packaging and migrated to the simulant. When the LLDPE and LDPE final packaging were emptied, the simulant was preserved. Samples of simulant (300 mg) treated with simulated solar irradiation or thermic shock in LLDPE and LDPE final packaging containers were analyzed by HS-SPME/GC-MS. No substances related to the polymeric materials were detected within the formulation.

### 4.3. Simulant Characterization

**Figure 5:**
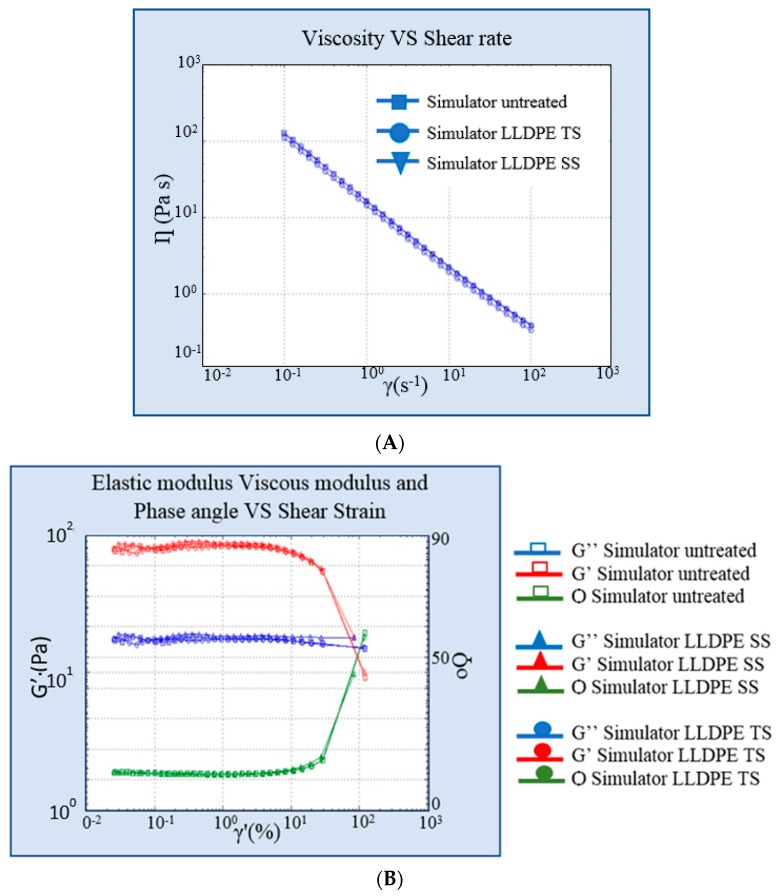
(**A**) Viscosity curve of simulant in linear low-density polyethylene (LLDPE) vs. untreated simulant. (**B**) Elastic and viscous modulus curves of formulation in LLDPE vs. untreated simulant. SS: simulated solar irradiation, TS: thermal shock.

**Figure 6:**
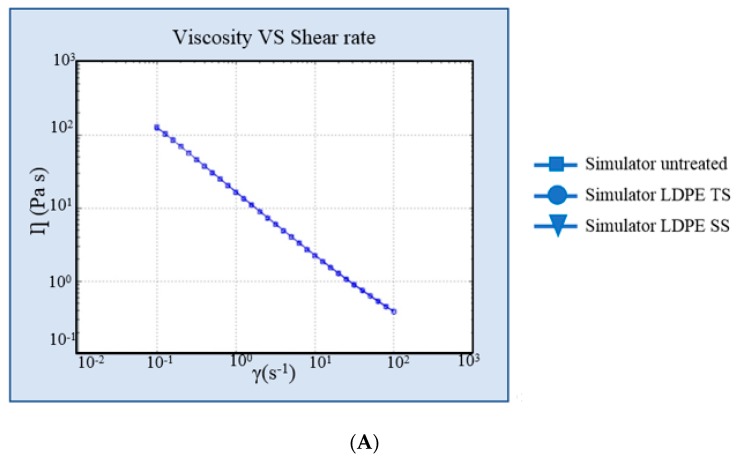

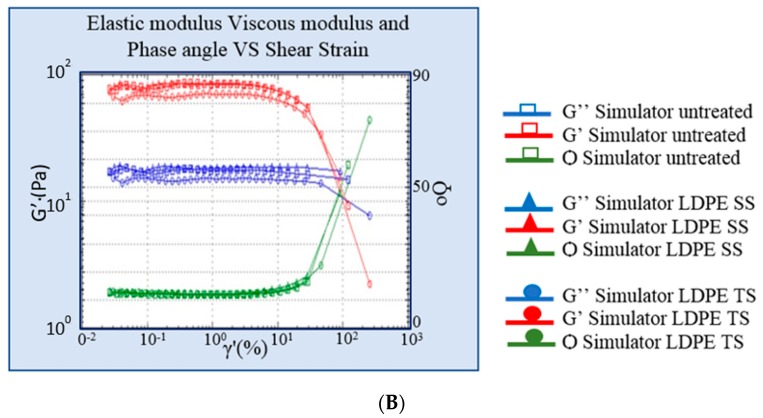
(**A**) Viscosity curve of simulant in low-density polyethylene (LDPE) vs. untreated simulant. (**B**) Elastic and viscous modulus curves of simulant in LDPE vs. untreated simulant. SS: simulated solar irradiation, TS: thermal shock.

pH measurements and organoleptic properties analysis showed no changes between untreated simulant and treated simulant in the LLDPE or LDPE single dose container. The evaluation of the rheological properties of the simulant underlined that the two different types of materials did not change in terms of viscosity or the rheological behavior of the content. The viscosity of the simulant contained in the two types of plastic material was unaltered after both treatments. Figure 5 and Figure 6 show the viscosity curve and elastic and viscous modulus curves of the simulant in LLDPE and LDPE vs. untreated simulant.

## 5. Discussion

In this study, we investigated the differences between standardized samples and those obtained from final packaging samples. Evaluations of final packaging provided information about the materials after manufacturing processes and the possible interactions between the packaging and the content. Some interactions between packaging and content could occur during in-use conditions. The literature reports a correlation between the changes in the mechanical behavior and changes in the barrier properties, resulting in changes in quality, efficacy, and safety of the products [28]. Therefore, we evaluated the mechanical characteristics.

In the first place, thickness testing was performed on ISO and dog-bone shaped specimens. Among the different polymer processing techniques used to produce plastic packaging with the desired size, shape, and characteristics, injection molding is the most used in the polymer industry. A critical aspect of the replication is the high precision involved [29,30]. Wall thickness testing allowed us to determine if the plastic material was uniform or, during the blow-molding process, the uniformity was not optimized, compromising the quality of the product [31].

Table 4 shows the thickness data mean obtained on ISO 527 and dog-bone shaped specimens expressed as a mean ± % standard deviation. Differences in the thickness values between the two materials are displayed. Those differences are related to the intrinsic characteristics of the polymer and the manufacturing process. ISO specimens are created via the high pressure injection of the melted polymeric pellets in a standard dimension mold. No information about the injection region was specified on ISO 527; thus, ISO specimens were created by injection at the bottom and injection at the side of the mold. Specimens produced by injection at the bottom have vertical polymeric chain orientation (LLDPE and LDPE vertical) and specimens produced by injection at the side have a horizontal polymeric chains orientation (LLDPE and LDPE horizontal). The thickness of the LLDPE and LDPE ISO specimens were homogenous, between 4.1 to 4.2 mm. This homogeneity, however, was hard to attain with specimens obtained from the final packaging, where the thickness is regulated by the packaging production and destination. The mean LLDPE and LDPE single dose container thicknesses were 610.4 and 565.6 µm, respectively.

**Figure 7:**
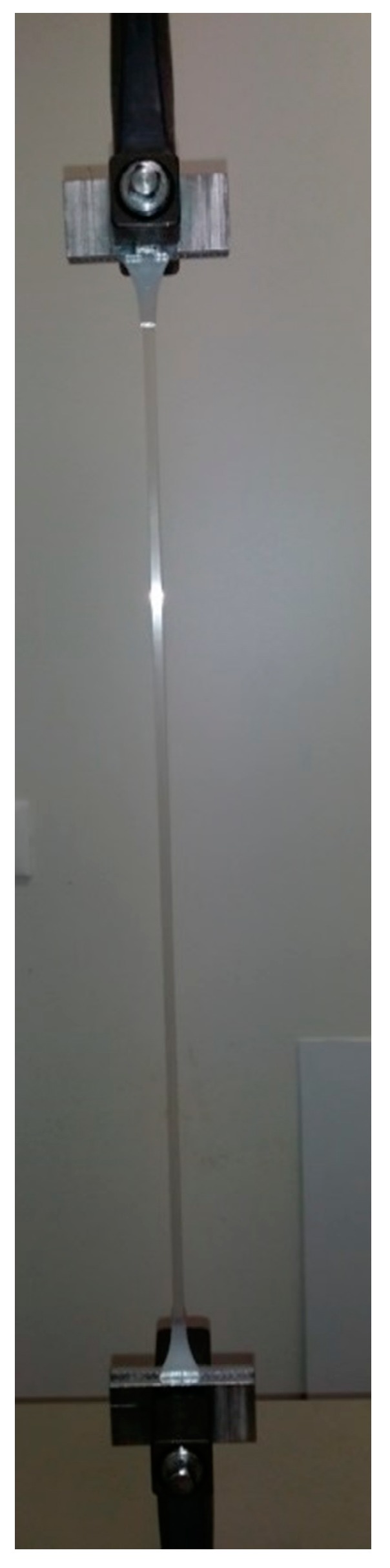
ISO-LLDPE (linear low-density polyethylene) specimen during tensile test.

Table 5 shows the tensile test data obtained from ISO and dog-bone shaped specimens expressed as a mean ± % standard deviation. Obtaining Horizontal and Vertical LLDPE and LDPE stress-strain values at yield was not possible because the stress-strain profile obtained from the tensile test was not clearly detectable. Similarly, it was not possible to obtain Horizontal and Vertical LLDPE stress-strain values at break since the material elongation was greater than the instrument tensile stroke in the set conditions (10 mm/min), showing the lack of versatility of the standardized specimen. Figure 7 shows the ISO-LLDPE profile as an example.

Data obtained from the LLDPE and LDPE dog-bone shaped specimens tensile testing highlighted the different mechanical behavior in terms of elongation percentage and stress (MPa) between the two polymers. The values of stress at yield point and at break had standard deviations within a range of 10%, demonstrating high homogeneity. Starting from this low standard deviation, those values can therefore be considered as significant parameters for evaluating possible changes in the mechanical properties of plastic material considered before or after the treatment and/or contact with a simulant. For the other parameters, like strain and the angular coefficient, a higher standard deviation was found, so these parameters were not further considered as relevant to underline possible changes undergone by the material after stress.

Afterward, we evaluated the plastic material mechanical properties by mimicking the in-use conditions. This step was not possible with the ISO samples. The LLDPE and LDPE single dose container, both empty or filled with the simulant, were exposed to accelerated stability testing and analyzed using the tensile test. The use of silicones in a broad range of products has exponentially increased; thus, the simulant contained a silicon component in order to simulate the performance and the interaction of a formulation commercially available [21,22]. A future work could propose different simulants to reflect a complete range of formulation properties.

Figure 1 shows a graph representing the trend in the yield point values and the statistical analysis completed using the Mann-Whitney test with a 95% confidence interval on LLDPE and LDPE dog-bone shaped specimens before and after the treatments. Simulated solar radiation did not induce significant changes in the LLDPE polymer mechanical characteristics. No significant variations in the mechanical properties were recorded for the polymer in contact with simulant after the treatment. Otherwise, results for the LDPE polymer showed that simulated solar radiation induced statistically significant changes in the mechanical characteristics of this polymer and induced some interactions between the formulation and the container detectable at the level of alterations of the mechanical properties of packaging composed of this polymer.

Conversely, thermal shock treatment significantly influenced the mechanical behavior of both empty polymers. Furthermore, in both polymers, the simulant significantly interacted with the container when subjected to thermal shock.

### 5.1. Extractable Characterization

Many protocols used to obtain the extractable profile of plastic material have been studied. All these protocols focus on the raw material (pellets) but no information was found about the extractable profile of the plastic material obtained from the final packaging. As explained previously, during the manufacturing process, some additives are used, which can create a complex extractable profile. In this part of the study, the volatile organic extractable profile of the plastic materials obtained from untreated final packaging was investigated. Multiple extraction processes and extraction solvents were used to detect the technique able to identify the major class of components. This work group, previously, determined three different categories of detectable components, as reported in Table 6: (1) compounds associated with the initial ingredients, (2) impurities related to processing, and (3) degradation products of polymers [27].

A preliminary study was performed on the untreated LLDPE and LDPE. Multiple extraction processes were evaluated in relation to the plastic materials investigated and according the literature [27]: Sonication, Sealed Vessel, Soxhlet, and HS-SPME. Figure 3 and Figure 4 show the GC/MS chromatograms of all the extraction methods of untreated LLDPE and LDPE single dose containers. Chromatographic analyses showed that HS-SPME and Soxhlet extractions provide complete insight into all the major organic extracts for the analyzed materials. The HS-SPME extraction contained the same extracts as n the Soxhlet extraction, but with higher concentrations, so the methods were optimized. HS-SPME extraction was defined as the best method for performing the controlled extraction of volatile organic extracts in plastic material obtained from the final packaging.

LLDPE and LDPE single dose containers, both empty and filled with simulant, were characterized after accelerated stability testing. The extract profiles of the treated samples were obtained with HSSPME extraction and the extracts were analyzed by GC/MS.

Table 7 shows the percent area of each class of extracted substance. In filled samples after simulated solar irradiation or thermal shock, substances closely related to the simulant were detected in the extractable profile of the plastic material. These substances were identified as Decamethylcyclopentasiloxane-Cyclomethicone D5 and *Bis*(2-ethylhexyl) carbonate. They represented nearly 95–96% of the total extracted compounds (Figure 4). To exclude the possibility that the identified substances simply remained on the surface of the polymers in a non-efficient washing system, LLDPE and LDPE samples from the final packaging were placed in the simulant for 30 min. Then, they were cleaned with the washing method used for all the samples. An extractable profile, with the selected test technique (HS-SPME), was performed. After GC/MS analysis, no traces of the substances associated with the simulant were found. This could strongly indicate the efficacy of the washing system used, confirming that some simulant substances could have been adsorbed by the packaging material examined in specific stress conditions.

In addition to assessing the extractable profiles, a first screening was used to evaluate the substances leached from the final packaging that migrated to the simulant. When the LLDPE and LDPE final packaging were emptied, the simulator was preserved. Samples of simulant (300 mg), treated with simulated solar irradiation or thermic shock in LLDPE and LDPE final packaging containers, were analyzed by HS-SPME/GC-MS. No substances related to the polymeric materials were detected within the formulation. The limit of detection is one of the most important topics in extractable and leachable analysis. The Safety Concern Threshold (SCT) below 0.15 μg/day has been defined as the leachable threshold that would present negligible safety concerns from possible carcinogenic to noncarcinogenic toxic effects [32]. Results obtained from this study suggest that the phthalate levels would be below the SCT level of 0.15 μg/day. This work was largely qualitative. Future studies will focus on quantifying the leachable amount according to the safety assessment depending on the product category and exposure levels during use.

### 5.2. Simulant Characterization

pH measurements and organoleptic properties analysis showed no changes between the untreated and treated simulants containing LLDPE/LDPE single dose containers. Figure 5 and Figure 6 demonstrate the viscosity curve and the elastic and viscous modulus curves of the simulant after simulated solar testing in LLDPE and LDPE vs. the untreated simulant. The evaluation of the rheological properties of the simulant through the rheometer showed that either the viscosity or the rheological behavior of the content of the two different types of materials did not change. The viscosity of the simulant contained in the two types of plastic material were unaltered after both treatments.

## 6. Conclusions

In this study, we investigated the differences between standardized samples and final packaging samples. We compared ISO and dog-bone shaped specimens. ISO specimens were homogeneous in terms of thickness, but they could not be used to analyze the final packaging. Instead, the dog-bone shaped specimens used in this work were successfully employed for all packaging shapes, including small packages as single units.

Among the mechanical parameters, the yield stress at and the stress at break point were better indicators for evaluating any changes in the material characteristics before and after treatments.

From results obtained in this study, the thermal shock is the better stress condition for evaluating LLDPE packaging mechanical properties and the possible interaction between content and container. Otherwise, results for the LDPE polymer showed that both simulated solar radiation and thermal shock treatment induced statistically significant changes in the mechanical characteristics of this polymer. Both induced some interactions between the formulation and the container detectable at the level of alterations of the mechanical properties of packaging composed of this polymer.

A preliminary study on the untreated LLDPE and LDPE was performed by multiple extraction processes according to the literature [24]. HS-SPME extracts provided complete insight into all the predominant volatile organic extracts. HS-SPME was selected as the test method to perform the successive controlled extraction studies. Analysis for both untreated and treated empty polymers showed that the largest percentage of compounds extracted are associated with the polymer degradation products. Instead, in both polymers that contacted the simulant after treatments, substances closely related to the simulant were detected at relatively high levels. These substances were identified as Cyclopentasiloxane and *Bis*(2-ethylhexyl) carbonate. The migration of product components represents an important factor for packaging quality and safety for human health. In the food industry, regulations for materials and packaging expected to contact food are in place to ensure constituents that could affect human health are not transferred. The food approach may also be used in the cosmetic industry.

This work underlined the importance of correctly studying the packaging material in relation to the content, to be able to detect possible interactions.

## Figures and Tables

**Table 1:** Qualitative and quantitative simulant composition used to fill the low-density polyethylene (LDPE) and linear low-density polyethylene (LLDPE) single dose containers.

Phase	Ingredient Name	International Nomenclature of Cosmetic Ingredients (INCI Name)	%
A	Xanthan gum	Xanthan gum	0.8
	pH 10 buffer solution (FUI XII Ed.) *	37% hydrochloric acid, borax, sodium hydroxide	59.2
B	Tegosoft DEC	Diethylhexyl carbonate	15
	Abil Care XL 80	Bis-PEG/PPG-20/5 PEG/PPG-20/5 Dimethicone; Methoxy PEG/PPG-25/4 Dimethicone; Caprylic/Capric Triglyceride	5
	Dimethicone KF995	Dimethicone	20

* pH 10 buffer solution (F.U.I XII Ed.) composition: 19.07 g borax, 1.4 mL hydrochloric acid 37%, 4 g sodium hydroxide pellet in 1000 mL purified water, and adjusting pH, if needed.

**Table 2:** Extraction methods specification.

Extraction Methods	Tested Article	Condition	Specification
Sonication	500 mg	150 mL in buffer solution pH 2 and pH 10	-
Sealed Vessel	500 mg	10 mL 1:1 isopropanol/water 55 °C for 3 days	-
Soxhlet	500 mg	150 mL Hexane 140 °C for 30 min	Soxtherm/Multistat Rapid Soxhlet Extraction System (Gerhardt)
HS-SPME	500 mg	Incubation temperature: 90 °C, Extraction time: 60 min	Headspace-mode, Fiber 100 μm Polydimethylsiloxane (PDMS), Supelco

**Table 3:** Operating parameters of gas chromatography-mass spectrometry (GC/MS) analysis.

Operating Parameters	Organic Extracts	Headspace (HS-SPME)
Column	Restek capillary column Rtx-5MS 30 m × 0.25 mm ID × 0.25 µm	Restek capillary column Rtx-5MS 30 m × 0.25 mm ID × 0.25 µm
Oven Program	Start at 50 °C, hold for 1 min; ramp 12 °C/min to 315 °C, hold for 16 min	Start at 60 °C, hold for 4.5 min; ramp 20 °C/min to 280 °C, hold for 5 min
Injector	CT Split/Splitless 300 °C Split flow 10 mL/min, split ratio 1:10	PTV Splitless 250 °C Splitless time 4.5 min
Injection	Split, 1 µL	-
Carrier Gas	Helium, 1 mL/min constant flow	Helium, 1 mL/min constant flow
MS Transfer line temperature	290 °C	270 °C
MS Detection Details	70 eV (+EI) Ion source 250 °C Mass range 35–650 amu Scan rate 803.7 amu/s	70 eV (+EI) Ion source 250 °C Mass range 50–650 amu Scan rate 870 amu/s

**Table 4:** Thickness data mean obtained on ISO 527 specimens and dog-bone shaped specimens made of linear low-density polyethylene (LLDPE) and low-density polyethylene (LDPE) expressed as a mean ± % standard deviation.

LLDPE (H-ISO)	LLDPE (V-ISO)	LDPE (H-ISO)	LDPE (V-ISO)	LLDPE	LDPE
Thickness (mm)				Thickness (µm)	
4.1 (±1.69)	4.2 (±1.01)	4.2 (±0.79)	4.1 (±0.92)	610.4 (±1.73)	565.6 (±1.17)

**Table 5:** Tensile test data obtained from ISO and dog-bone shaped specimens made of linear low-density polyethylene (LLDPE) and low-density polyethylene (LDPE) expressed as a mean ± %standard deviation.

Parameter	Sample			
	LDPE (H-ISO)	LDPE (V-ISO)	LLDPE	LDPE
Tensile strength (σM) = yield stress (σy) (MPa)	-	-	7.5 (±7.97)	6.7 (±4.29)
Tensile strain at yield (εy) = (εM) (%)	-	-	9.4 (±17.02)	n.d.
Angular coefficient linear portion	-	-	123.6 (±16.87)	110.7 (±9.51)
Tensile stress at break (σB) (MPa)	10.0 (±3.00)	83.5 (±2.96)	10.8 (±8.53)	7.7 (±9.15)
Tensile strain at break (εtB) (%)	9.8 (±4.41)	83.1 (±2.68)	290.0 (±12.81)	86.4 (±25.44)

- Data not obtained. n.d. not determined.

**Table 6:** Organic extractable profile extracted using the Head Space Solid Phase microextraction (HS-SPME) method and analyzed by gas chromatography-mass spectrometry (GC/MS) expressed as a percentage of linear low-density polyethylene (LLDPE) and low-density polyethylene (LDPE) from empty untreated final packaging divided into three different categories: (1) compounds associated with the initial ingredients, (2) impurities related to processing, and (3) degradation products of polymers.

Compound Categories	Identification	CAS NR (Chemical Abstract Service Number)	Chemical Formula	Molecular Weight	LLDPE (% area)	LDPE (% area)
1	2,4-Di-t-butyl phenol	96-76-4	C_14_H_22_O	206	0.11	0.26
	Hexadecanamide	629-54-9	C_16_H_33_NO	255	traces	traces
	9-Octadecenamide, (*Z*)-	301-02-0	C_18_H_35_NO	281	traces	traces
	Hexadecyl 2-ethylhexanoate	59130-69-7	C_24_H_48_O_2_	368	12.39	traces
	Diisobutyl phthalate	84-69-5	C_16_H_22_O_4_	278	2.26	5.05
	Dibutyl phthalate	84-74-2	C_16_H_22_O_4_	278	2.80	3.81
	Irganox 1076	2082-79-3	C_35_H_62_O_3_	530	11.94	3.82
	Diisooctyl phthalate	131-20-4	C_24_H_38_O_4_	390	0.32	5.44
2	Myristyl myristate	3234-85-3	C_28_H_56_O_2_	424	traces	traces
	Octinoxate	5466-77-3	C_18_H_26_O_3_	290	3.01	1.47
3	Aliphatic hydrocarbons *	-	-	-	57.14	61.56
	Olefins *	-	-	-	7.39	10.26

* class of compounds.

**Table 7:** Percent area of substances extracted with the Head Space Solid Phase microextraction (HS-SPME) method and analyzed by GC/MS from linear low-density polyethylene (LLDPE) and low-density polyethylene (LDPE) single dose containers, both empty and filled with simulant, after simulated solar irradiation (SS) and thermal shock (TS), compared with untreated samples.

Compound Categories	LLDPE (% area)					LDPE (% area)				
	Empty	Empty		Filled		Empty	Empty		Filled	
	Untreated	SS	TS	SS	TS	Untreated	SS	TS	SS	TS
Compounds associated with the initial ingredients	17.4	<0.01	2.4	0.04	0.2	18.1	<0.01	2.1	<0.01	0.8
Compounds related to processing	18.0	1.3	17.5	3.4	3.3	10.1	0.8	14.7	4.0	3.1
Degradation products of polymers	64.52	98.7	80.0	1.8	1.9	71.82	99.2	83.2	1.1	1.0
Compounds absorbed from simulant	**-**	**-**	**-**	94.8	94.7	**-**	**-**	**-**	94.8	95.7
